# Internal Fixation With Cannulated Screws for Stable Femoral Neck Fractures in High-Risk Patients: Good Clinical Outcomes and Evaluation of Postoperative Complications

**DOI:** 10.7759/cureus.71767

**Published:** 2024-10-18

**Authors:** Dimitrios Giotis, Christos Konstantinidis, Sotiris Plakoutsis, Christos Kotsias, Alkisti Konstantinou, Dimitrios Tsiampas, Dimitrios Vardakas, Vasileios Panagiotopoulos

**Affiliations:** 1 Orthopaedic Department, General Hospital of Ioannina "G. Hatzikosta", Ioannina, GRC

**Keywords:** cannulated screws, complications, femoral neck fractures, high-risk patients, internal fixation

## Abstract

This study aimed to investigate the clinical outcome and the postoperative complications after internal fixation with cannulated screws of stable femoral neck fractures (FNFs) in high-risk patients. A total of 76 patients (mean age 70.11 ± 9.83 years) with stable FNFs participated in the study. All patients underwent fixation with two or three cannulated screws (percutaneous). Postoperatively, they were evaluated with the Harris Hip Score (HHS), while parameters regarding other possible comorbidities or delayed complications were also assessed. Regarding the HHS, more than 75% of patients presented satisfactory results at the last follow-up. No wound infection or hip dislocation was reported. In seven cases (9.21%), complications were observed such as nonunion or osteonecrosis of the femoral head, which were treated successfully with a revision surgery. Conclusively, screw fixation in high-risk patients could be an effective, minimally invasive procedure for the treatment of stable FNFs with satisfactory clinical results, a low revision rate, and potential to return to pre-injury activities.

## Introduction

The incidence of intracapsular femoral neck fractures (FNFs) presents an annual increased tendency because of the aging of the population [1]. They constitute 40-50% of all hip fractures [2]. The classification of Garden, which is still commonly used worldwide, categorizes FNFs as stable (type 1 and 2) and unstable (type 3 and 4) [3]. Stable FNFs are impacted or nondisplaced and represent 20-30% of all FNFs, whereas over 70% are unstable and displaced [4].

In elderly patients with displaced FNFs, the most widely used operation method is hip hemiarthroplasty (HA), which is associated with good clinical outcomes [5,6]. However, in these patients, when FNFs are nondisplaced, the treatment is still controversial [7-12]. Several studies report that elderly patients with nondisplaced FNFs, which were treated primarily with hemiarthroplasty, displayed satisfactory clinical results presenting less pain, higher quality of life, and a lower risk of reoperation [7,9,10]. Moreover, those who were treated with internal fixation (IF) demonstrated poor functional outcomes, severe postoperative (post-op) complications, higher rate of reoperation, and impairment of quality of life [9,10].

On the contrary, other studies advocate that IF with cannulated screws is the method of choice for nondisplaced FNFs in elderly patients, reporting that it is less invasive, requires shorter operating time, and presents lower intraoperative blood loss and lower risk of post-op pulmonary complications and surgical site infections, as compared to hemiarthroplasty [13,14].

Furthermore, most of elderly patients suffer from other major or minor comorbidities. In cases of a hip fracture in these patients, the health status frequently deteriorates even with dramatic results. Indeed, high rates of morbidity and mortality have been observed after this kind of trauma [12,14]. The purpose of this study was to investigate the clinical outcome and the post-op complications after IF with cannulated screws of stable FNFs in the elderly or in patients with comorbidities. We hypothesized that IF in these high-risk patients would be an effective procedure with a good clinical outcome, a low revision rate, and potential to return to pre-injury functional level.

## Materials and methods

Patients

From January 2015 to December 2018, 76 patients with stable FNFs, who were admitted to our hospital, were retrospectively evaluated. The mean age at surgery was 70.11 ± 9.83 years (range 66-84 years). All the participants were over 65 years old, with or without comorbidities, who had sustained a nondisplaced FNF after low-energy trauma that was treated with IF with cannulated screws. All of them were considered high-risk patients due to their increased age or coexisting comorbidities. On the contrary, patients who were less than 65 years old, had insufficient pre-fracture mobilization capacity or hip osteoarthritis, or had sustained unstable FNFs or intertrochanteric, periprosthetic, pathologic, or concomitant pelvic fractures were excluded from the study. In addition, patients with stable FNFs who were treated with other methods, such as hemiarthroplasty, were not included in the study.

**Figure 1:**
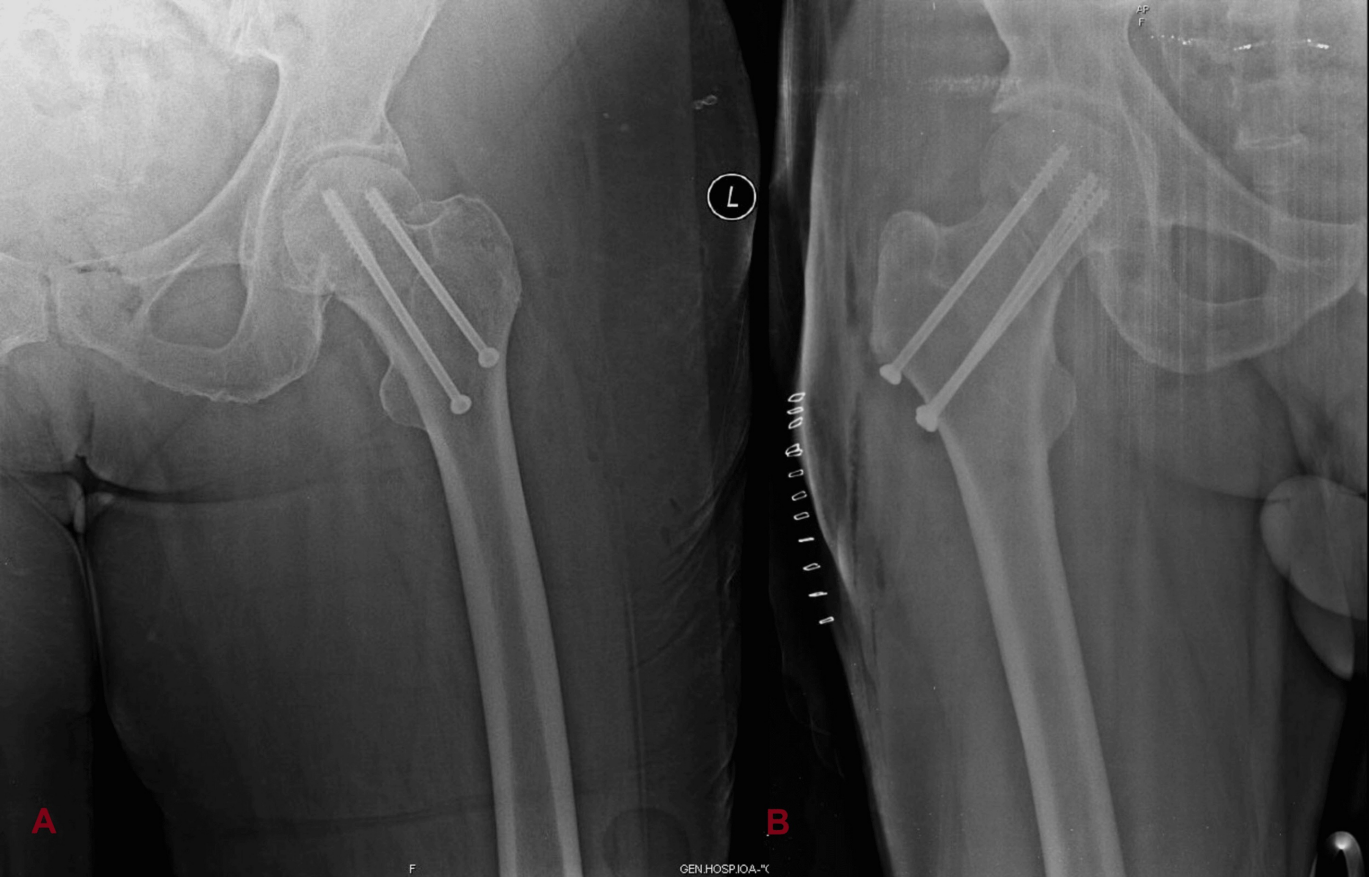
X-rays demonstrating the fixation of FNFs with cannulated screws A: Use of two screws. B: Use of three screws. FNF, femoral neck fracture

Thus, according to Garden’s classification for FNFs, patients with FNFs type 1 and 2, which are considered nondisplaced fractures, were included in the study, whereas those with type 3 and 4, which are considered displaced fractures, were excluded from the study [3]. Furthermore, for all the patients who underwent IF, two (percutaneous) or three 6.5 mm cancellous cannulated screws were used (Figure 1).

Screw fixation was performed on a traction table under fluoroscopy with a minimally invasive procedure, avoiding capsulotomies or hemarthrosis aspirations. Additionally, the initial diagnosis of the hip fracture was conducted with radiographs or even CT scans in controversial cases.

The current study was reviewed and approved by the institutional review board of our hospital. Data were collected from all patients who agreed to participate by signing informed consent forms in accordance with the Helsinki Declaration of Principles of 1964, as revised in 2008 [15].

Assessment methods

The patients were evaluated with Harris Hip Score (HHS), and all clinical data were collected at the last follow-up. The mean post-op follow-up time was 3.4 ± 2.7 years. Apart from hip joint function, parameters regarding other possible comorbidities or delayed complications were also assessed. Thus, incidence of surgical complications, including avascular necrosis of the femoral head, nonunion, implant failure, superficial or deep wound infection, periprosthetic fracture, and dislocation, were recorded, along with revision surgery rate due to complications of the primary procedure such as mechanical failure of fixation (screw backing out of the femoral head). Likewise, the mortality rate, as a consequence of surgical complications or not, was also adopted.

Moreover, perioperative factors including length of hospital stay, intraoperative blood loss, and blood transfusion rate were also recorded. Concerning the HHS, the maximum score was 100. A score of 90-100 was regarded as “excellent,” 80-89 as “good,” 70-79 as “fair,” and <70 as “poor.”

The statistical analysis of all data was conducted using the IBM SPSS Statistics, version 26.0 (IBM Corp., Armonk, NY), including a descriptive analysis of data that involved distribution, measures of central tendency, and measures of variability.

## Results

The male-to-female ratio was 1:1.71. Specifically, 28 patients were male and 48 were female. The most common mechanism of injury was a fall from a standing height (64.47% [n=49]). The injury-surgery interval was 22.2 ± 7.3 hours. The perioperative parameters that were recorded during patients' hospital stay are presented in Table 1. In the two patients who were in need of blood transfusion, there was a hemoglobin level drop from 10.9 g/dL and 11.1 g/dL for each patient preoperatively to 8.6 g/dL and 8.5 g/dL post-op, respectively.

**Table 1:** Patients' baseline characteristics (mean ± SD) / perioperative parameters

Parameters	Total (n=76)
Age (years)	70.11 ± 9.83
Gender	
Male (%)	28 (36.84)
Female (%)	48 (63.16)
Side	
Right (%)	40 (52.63)
Left (%)	36 (47.37)
Garden classification	
1 (%)	30 (39.47)
2 (%)	46 (60.53)
Perioperative parameters	
Length of hospital stay (days)	8.13 ± 1.88
Intraoperative blood loss (mL)	46.82 ± 18.25
Blood transfusion rate	1/38

Regarding the HHS at the last follow-up, 10 patients (13.16%) were categorized as excellent, 21 patients (27.63%) as good, 27 (35.53%) as fair, and 18 (23.68%) as poor, whereas the mean HHS was 78.4 ± 15.3. The cumulative percentage of participants who presented excellent, good, or fair results in HHS is considered satisfactory, taking into account that the participants were elderly or had comorbidities.

In Table 2, patients' comorbidities as recorded with their admission to the hospital are demonstrated. In terms of post-op complications, no case of superficial or deep wound infection was reported. Similarly, no case of hip dislocation was observed. On the contrary, three patients (3.95%) experienced osteonecrosis of the femoral head, two patients (2.63%) nonunion, one patient (1.32%) mechanical failure with screw mobilization, and another one (1.32%) subtrochanteric fracture in the follow-up period. Generally, complications related to the primary procedure was found in seven participants (9.21%), who were reoperated (Table 3).

**Table 2:** Comorbidities reported among the examined patients COPD, chronic obstructive pulmonary disease

Patients' comorbidities	n (%)
Hypertension	57 (75)
Heart disease	28 (36.84)
Depression	21 (27.63)
Dyslipidemia	20 (26.32)
Diabetes mellitus	18 (23.68)
Gouty arthritis	10 (13.16)
Rheumatoid arthritis	9 (11.84)
Hypothyroidism	8 (10.53)
COPD	6 (7.89)
Cancer	6 (7.89)
Alzheimer's disease	4 (5.26)
Parkinson's disease	3 (3.95)

**Table 3:** Postoperative complications / revision surgery / mortality

	n (%)
Femoral head osteonecrosis	3 (3.95)
Nonunion	2 (2.63)
Screw cutout	1 (1.32)
Subtrochanteric fracture	1 (1.32)
Total post-op complications	7 (9.21)
Reoperations	7 (9.21)
Mortality (total)	12 (15.79)

The mean time between primary operation and ischemic necrosis of the femoral head for the three cases in our study was 6.33 ± 2.52 months (range 4-9). In all these patients, a hemiarthroplasty was conducted. Furthermore, in the two patients with nonunion, the time between IF and nonunion was five and six months, respectively, whereas for the case with the mechanical failure the time between IF and screw cutout was two months. All the patients with nonunion and screw cutout were also treated with hemiarthroplasty. The subtrochanteric fracture was noted at three months postoperatively and was treated with intramedullary fixation with long gamma nail. Lastly, the total mortality rate was 15.79% (12/76), which included patients who had died from the first post-op day until the last follow-up. The main causes of death were cardiac arrest, heart failure, ictus cerebri, cancer, or cerebral hemorrhage.

## Discussion

In the current study, it was demonstrated that IF of nondisplaced FNFs in high-risk patients due to increased age or coexisting comorbidities is an effective, minimally invasive technique, which could provide satisfactory clinical outcomes and potential to return to pre-injury activities as it is related to fewer surgical complications and a low revision rate.

We report good clinical results regarding pain and hip joint function as extracted by HHS analysis of the last follow-up. A similar good clinical outcome was also reported in the systematic review of Oñativia et al. [8]. Yih-Shiunn et al. also noted satisfactory clinical results, displaying a mean HHS of 82.6 after a mean 2.9-year follow-up time interval [16].

Concerning the perioperative parameters, Chen et al. displayed, similar to our report, intraoperative blood loss after IF in stable FNFs [12]. Comparable results with our survey were reported in this article, concerning also the parameters “blood transfusion rate” and “length of hospital stay” [12]. On the contrary, increased intraoperative blood loss was reported by Reina et al. after IF [11]. Especially, as far as the factor “length of hospital stay” is concerned, in literature it varies from six to 17 days after IF in intracapsular hip fractures [8].

Regarding the reoperation rate after IF for nondisplaced FNFs and the percentage of conversion to hip arthroplasty after failure of IF, ranges from 8% to 19% and from 8% to 16% have been observed correspondingly [8]. Manohara et al. reported the lowest reoperation and conversion rates (conversion to hemiarthroplasty or arthroplasty), which was 8% for both rates [13]. Bjørgul and Reikerås noted the highest reoperation rate (19%) whereas the highest conversion rate was found by Chen et al. and Shimizu et al. [17-19]. Both studies present a conversion rate of 16.2% [18,19]. In our series, a reoperation rate of 9.21% was observed, which was among the lowest percentages reported in the literature [8]. Apart from the subtrochanteric fracture, all the other revision surgeries were converted to hemiarthroplasties. 

The complication rate that we found was also 9.21%, as all our post-op complications were revised surgically. This rate varies in the literature from 8% as reported by Manohara et al. to 21.5% as reported by Toh et al. [8,13,20]. These differences in complication rate in literature might be the result of patients' age, number of participants, or quality of the implemented implants, among others, that concerned each study [8]. Our results are consistent with these reports, but closer to the lowest rate, supporting that IF with screws is a safe method for approaching Garden 1 and 2 fractures even in elderly patients or patients with comorbidities. A similar outcome was presented by Bigoni et al. who signified a total complication rate of 8.9% after IF of stable intracapsular FNFs in the elderly. However, they reported that only 4.5% of these patients required hemiarthroplasty to recover a comparable quality of life with that prior to the fracture [14].

Furthermore, femoral head osteonecrosis and nonunion, as post-op complications, represent 2.5-13.7% and 3-11% of stable FNF IFs, respectively [8,13,20-23]. Concerning the post-op complications, peri-implant fracture, and mechanical failure such as screw cutout, they constitute 0.5-7.4% and 0-12.2% of nondisplaced FNFs treated with IF accordingly [8,13,18-20,23,24]. In our study, the percentages of these complications were again near to the lowest aforementioned levels. Several studies support that the degree of osteoporosis that patients with a hip fracture might present could affect fracture healing and post-op complication rate in general [25,26]. In particular, elderly patients, who are frequently osteoporotic, are at a higher risk of ischemic necrosis of the femoral head and nonunion due to poor fracture healing potential or of mechanical failures as a consequence of early loosening of the screws in the case of osteoporotic lateral cortex of the proximal femur [25,26]. In contrast, even the number or type of screws or the tridimensional implant placement may be factors associated with the fixation failure [27].

Moreover, we should highlight that no case of post-op superficial or deep infection was noticed in our patients. Similarly, a very low percentage or absence of infections after IF was reported by other articles [8,16,17]. As post-op infection could impair the health status variously, this might cause a detrimental effect on elderly patients' mortality [8,16,17].

We report a mortality rate of 15.79% after a mean follow-up of 3.4 years. Close to our outcome, Manohara et al. demonstrated a mortality rate of 18% with a mean follow-up of 3.25 years [13]. According to Oñativia et al., Kim et al., and Sikand et al., the mortality rate in patients who have undergone fixation with femoral screws for stable FNFs varies from 3% to 10% for the first six months, 13-36% at one year, and 12-40% at two years [8,28,29]. Kain et al. demonstrated a 42% mortality rate but after a five-year follow-up [22]. It is regarded that patient's age at the time of injury could play a critical role not only for the short-term but also for the long-term mortality rate [8,10]. In parallel, it has been found that a high reoperation rate could lead to increased mortality [8]. 

There are several limitations of our study that should be acknowledged. First, this is a retrospective study conducted in a single medical center. In addition, there was neither a control group nor a group consisting of patients with stable FNFs who would have been treated with hemiarthroplasty to compare our results with. However, the aim of this article was not to compare but only to quote and assess the clinical outcome and the post-op complications, along with the reoperation and mortality rates after IF in high-risk patients with stable FNFs, as there is still a controversy in this area. Moreover, the inclusion of deaths in our study for the evaluation of mortality rate could have an effect on the complication rate. Therefore, for these patients, all data were collected at their last follow-up. Finally, we did not take into consideration that osteoporosis could affect the complication and subsequently the reoperation rate, and thus, no evaluation of participants' osteoporosis status was performed.

## Conclusions

Considering the fragility of elderly people or patients with comorbidities, we suggest that IF could be a safe and effective procedure for nondisplaced FNFs in these cases, with satisfactory clinical outcomes and potential to return to pre-injury activities. However, further prospective randomized controlled trials are required to establish the optimal approach for the treatment of stable FNFs in patients with a high-risk profile, in terms of functional activity, morbidity, and mortality.
